# AMY-101 as complement C3 inhibitor for periodontitis therapy: mechanisms, efficacy, and clinical translation

**DOI:** 10.3389/fimmu.2025.1587126

**Published:** 2025-04-29

**Authors:** Jialun Li, Zhi Xu, Wayne Nishio Ayre, Xiaohan Liu

**Affiliations:** ^1^ Department of Prosthodontics, School and Hospital of Stomatology, China Medical University, Shenyang, China; ^2^ Liaoning Provincial Key Laboratory of Oral Diseases, China Medical University, Shenyang, China; ^3^ Department of Otolaryngology, The Second Affiliated Hospital of Shenyang Medical College, Shenyang, China; ^4^ School of Dentistry, Cardiff University, Cardiff, United Kingdom

**Keywords:** AMY-101, periodontitis, complement system, complement C3, bone resorption, bone formation

## Abstract

Periodontitis is a chronic inflammatory disease characterized by gingival inflammation, alveolar bone resorption, and periodontal tissue destruction. Complement activation, particularly through the C3 component, plays a critical role in the inflammatory processes underlying periodontitis. AMY-101, a selective inhibitor of complement C3, has demonstrated significant potential in modulating complement activity and mitigating periodontal inflammation. This study comprehensively evaluates AMY-101’s effects through *in vitro*, preclinical, and clinical studies. Mechanistic investigations revealed that AMY-101 effectively suppresses pro-inflammatory cytokines and matrix metalloproteinases (MMPs), reducing tissue destruction. Preclinical models confirmed AMY-101’s ability to improve clinical parameters such as probing pocket depth, attachment loss, and bone preservation. Moreover, clinical trials demonstrated AMY-101’s safety and efficacy in reducing gingival inflammation and bleeding without serious adverse events. These findings highlight AMY-101’s therapeutic potential for periodontitis and broader applicability in other complement-driven inflammatory diseases.

## 1 Introduction

Periodontitis is a reversible inflammatory disease caused by biofilm accumulation and ecological dysregulation in the subgingival region around the teeth, characterized by gingival inflammation (1), periodontal pocket formation and alveolar bone resorption. Without proper treatment, this oral disease can lead to tooth loss (2), which can result in poor aesthetics, deterioration of masticatory function and reduced quality of life (3, 4). With the deeper understanding of the pathological mechanisms of periodontitis, new therapeutic strategies have been proposed and applied, among which AMY-101 (C3 inhibitor), as a novel complement inhibitor targeting its complement component (C3), has attracted much attention in recent years for its application in the treatment of periodontitis (5). Studies have shown that AMY-101 has good safety and efficacy in a variety of primate disease models. It can block the propagation and amplification of the complement cascade (6), thereby significantly reducing inflammation and destruction of periodontal tissues and improving periodontal health without drug toxicity (7, 8), which is promising for application in the treatment of periodontitis. Therefore, this paper provides an overview of the mechanisms, efficacy, and clinical translation of AMY-101 in the treatment of periodontitis.

## 2 The relationship between complement C3 and periodontitis

**Figure 1:**
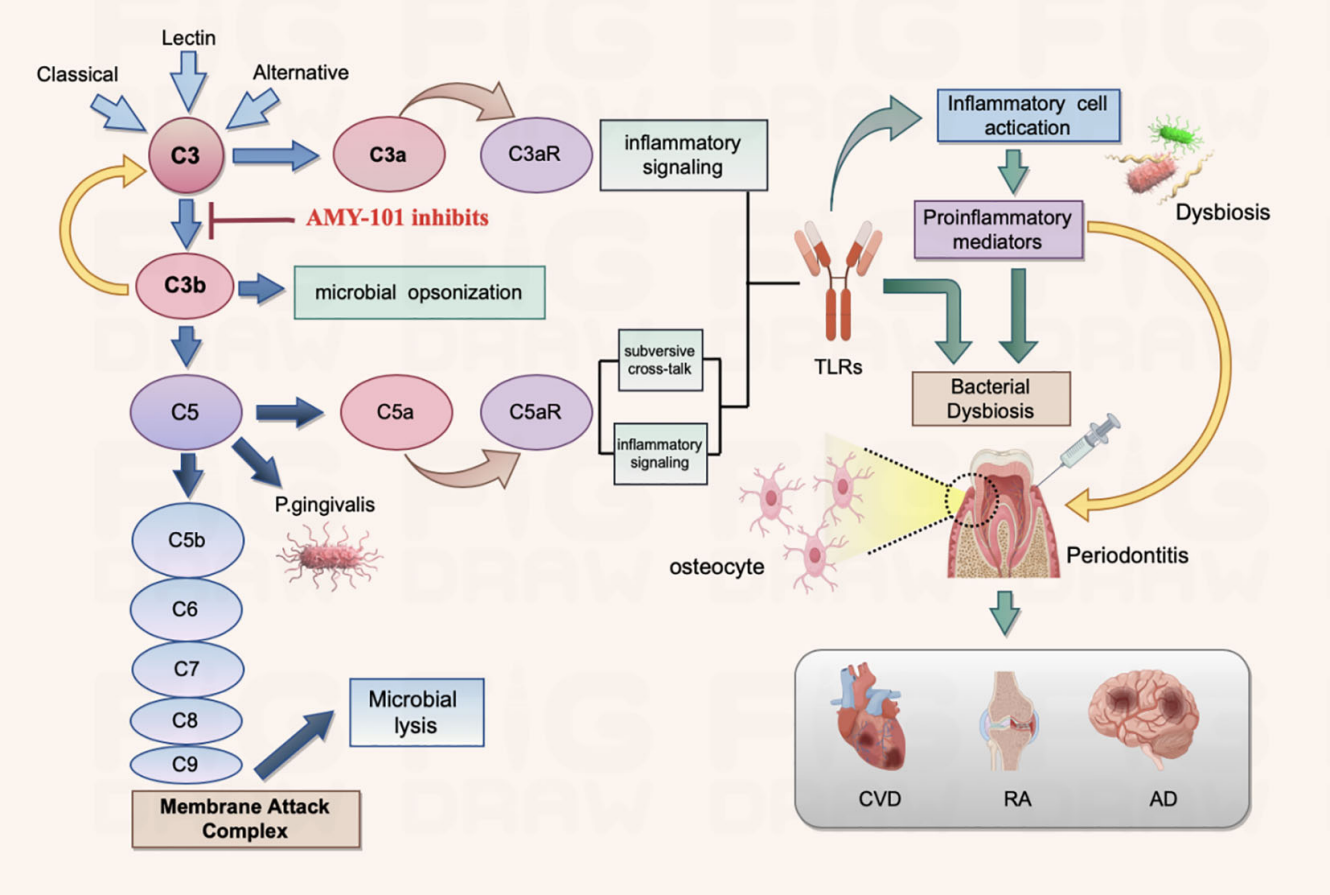
Schematic representation of the mechanisms involved in the regulation of periodontitis and other diseases by C3 complement and AMY-101.The central component of the complement system, C3, is activated through the classical, lectin, and alternative pathways, leading to its cleavage into C3b and C3a. C3b associates with C3 convertase to form C5 convertase, which subsequently cleaves C5 into C5a and C5b. C5b then sequentially binds to C6, C7, C8, and multiple C9 molecules, culminating in the assembly of the membrane attack complex (MAC). This complex integrates into the target cell membrane, creating pores that disrupt membrane integrity, ultimately causing cell lysis and death. Toll-like receptors (TLRs) upregulate the expression of C3aR and C5aR, leading to an inflammatory response that causes gingival tissue destruction and bone loss, ultimately resulting in periodontitis. These responses also reconstruct the commensal microbiota, exacerbating destructive inflammation and triggering other diseases, such as Alzheimer’s disease (AD), where the overactivation of C3a promotes neuroinflammation and neuronal damage, accelerating neurodegeneration (23); cardiovascular disease (CVD), where chronic inflammation induced by C3a causes endothelial damage and promotes atherosclerosis (31); and rheumatoid arthritis (RA), where C3 activation induces immune responses that aggravate joint inflammation and bone destruction (32).

The complement system is an important component of the innate immune system, a key immune locus that induces and regulates various immune and inflammatory functions (9), and plays a critical role in host defense against pathogen invasion, and maintenance of tissue homeostasis (10). The complement system is activated by the classical, alternative and lectin pathways (11), all of which converge at the level of C3, leading to cleavage of C3 to produce C3a and C3b (**Figure 1**) (12).

Although C3 activation has important biological functions in the immune response, it can be dysregulated or overactivated by host genetic or microbial virulence factors (13). At this point, the complement system can shift from homeostasis to pathological effects, leading to a wide range of inflammatory diseases such as periodontitis (3). In periodontitis, the balance between osteogenesis and bone resorption is disrupted (14), which is crucial for maintaining periodontal health (15). Complement activation products play a significant role in this process by inhibiting osteogenesis (16). C3a, a potent chemotactic factor, triggers the release of inflammatory mediators, recruits immune cells such as neutrophils and macrophages, and plays a crucial role in exacerbating inflammation (17). C3b, acting as an opsonin, marks pathogens, enhancing their clearance by phagocytic cells (18). In 2014, Matsuoka et al. (19) tested osteoblastogenesis-stimulating activity from osteoclast conditioned medium and identified C3 by continuous ion exchange chromatography. It was found that expression of the C3 gene was increased during osteoclastogenesis, that the cleavage product C3a was detected in osteoclasts by ELISA, and that daily administration of a C3aR inhibitor resulted in diminished bone formation. These results suggest that osteoclast-derived C3a plays a role in the transmission from bone resorption to formation in the absence of inflammation condition. Wingrove et al. demonstrated that C3 activated by the cysteine proteinase gingipain-1, generating chemotactic factors, and attracting neutrophils to the gingival lesion site, thus playing a role in the pathogenesis of periodontitis (20). Additionally, Wang et al. in a mouse periodontitis model found that C3a significantly upregulated IL-6 production through microbial stimulation and increased C3a receptor expression via ERK1/2 and JNK signaling pathways, thereby exacerbating inflammation and tissue destruction (21). Clinical studies have also demonstrated that concentrations of C3a in the gingival crevicular fluid of periodontitis patients are significantly elevated, correlating positively with disease severity (22). Additionally, Palmer et al. further investigated the function of complement C3b receptor 1 (CR1), highlighting its role in facilitating their clearance from the immune system (23).

In the complement activation cascade, C3b is involved in the formation of the C5 convertase, which cleaves C5 into C5a and C5b (24). C5a not only amplifies inflammatory responses but also facilitates tissue damage in periodontitis (25). It promotes osteoclast differentiation and activity, further contributing to bone resorption (26). Gan et al. developed a stiffness-tuned, ROS-sensitive hydrogel incorporating a C5a receptor antagonist that modulates macrophage antibacterial activity, offering new insights into therapeutic strategies for periodontitis treatment (27). These pathogens directly or indirectly activate the complement pathway by releasing specific enzymes and toxins, leading to the production of complement activation products such as C3a and C5a (16). Furthermore, Abe et al. also demonstrated the potential of using a C5a receptor (CD88) antagonist as a proof-of-concept for targeting complement in periodontitis, highlighting its ability to reduce inflammation and tissue damage (28).

Collectively, the interplay between C3a, C3b, and C5a is central to the inflammatory and immune responses observed in periodontitis (29). Clinical studies have demonstrated elevated levels of these complement activation products in the gingival crevicular fluid of patients with periodontitis, correlating with the severity of the disease (30). These findings highlight the importance of modulating the complement system, particularly through targeting C3a and C5a, as a therapeutic strategy for controlling inflammation and tissue damage in periodontal disease.

## 3 Overview of complement inhibitor AMY-101 (CP40)

AMY-101 (Cp40) is a cyclic C3 inhibitory peptide derived from the third-generation mitogenin analogue, featuring a cyclic structure that enhances its binding affinity and stability to C3 (33). This prevents the cleavage into C3a and C3b, thereby regulating the complement system (34). AMY-101 has a prolonged half-life, maintaining anti-inflammatory effects for approximately three months in animal models (35). In 2016, Mastellos et al. studied the effects of AMY-101 on non-human primates (NHPs) with chronic periodontitis. They administered AMY-101 subcutaneously at a dose of 4 mg/kg body weight once every 24 hours for 28 days. The treatment resulted in a significant reduction in periodontal pocket depth (PPD), an indicator of tissue destruction, demonstrating a marked improvement in periodontal health (36). Multiple trials above have affirmed the potential of AMY-101 as a new therapeutic strategy for a wide range of inflammatory conditions throughout the body, including periodontitis.

In preclinical studies, AMY-101 has been shown to significantly reduce inflammation and tissue damage and improve disease symptoms in various animal models (37). In transplant rejection models, Schmitz et al. observed that AMY-101 administration led to prolonged renal allograft survival in sensitized non-human primates, highlighting its potential in preventing antibody-mediated rejection (38). In nephropathy models, Mastaglio et al. reported the first case of COVID-19 treated with AMY-101, which successfully alleviated inflammation and improved clinical outcomes in a patient with severe SARS-CoV-2 infection (39). The reasons maybe that AMY-101 reduced the infiltration of specific inflammatory cells, such as neutrophils and macrophages, and decreased the levels of pro-inflammatory cytokines like IL-6 and TNF-α (40), mitigating tissue damage characterized by reduced edema, fibrosis, and cellular apoptosis, through the inhibition of complement activation (41). AMY-101 has also shown promising safety and efficacy in early clinical trials. In a clinical trial in patients with moderate to severe periodontitis, patients experienced significant improvements in periodontal health indicators such as periodontal pocket depth, gingival bleeding index and attachment loss following local injection or intra-pocket application of AMY-101, with no serious adverse events. In addition, AMY-101 has shown good safety and efficacy in clinical trials in other diseases such as Hemorrhagic Shock (42), further validating its potential for broad application as a complement inhibitor.

## 4 Mechanism of AMY-101 in periodontitis

AMY-101 is a promising therapeutic agent for periodontitis due to its targeted modulation of the complement system, specifically through selective C3 inhibition. In 2018, Bostanci et al. (43)administered AMY-101 topically to the maxillary gingival tissues of cynomolgus monkeys diagnosed with periodontitis and processed 45 samples of gingival fluid by FASP digestion and liquid chromatography-tandem mass spectrometry (LC-MS/MS) analysis, and found that several proteins involved in the complement activation, alternative pathway were down-regulated at 6 weeks by AMY-101, which effectively reduced the level of periodontal inflammation. It was found that several proteins involved in the “complement activation, alternative pathway” were down-regulated by AMY-101 at 6 weeks, which effectively reduced the level of periodontal inflammation. In 2017, Wu et al. concluded that IL-6 increased osteoblast-mediated osteoclast differentiation through activation of JAK2 and RANKL by finding that IL-6 and RANKL were stimulated in serum 3-7 days after orthognathic surgery, and RANKL expression was enhanced at both the mRNA and protein levels, which ultimately led to the conclusion that IL-6 increased osteoblast-mediated osteoclast differentiation through activation of JAK2 and RANKL (44).Wang et al. demonstrated that C3a cooperated with microbial stimuli that upregulated C3a receptor expression in an ERK1/2- and JNK-dependent manner to upregulate IL-6 production, so it can be suggested that AMY-101, as a C3 blocker, is closely related to osteoblast differentiation (21). These findings above suggest that AMY-101’s anti-inflammatory mechanism is essential for controlling the sustained inflammation that underlies periodontitis progression.

AMY-101 not only reduces inflammation but also plays a crucial role in preserving bone structure by modulating the RANKL/OPG signaling pathway (45), which regulates osteoclast activity and bone resorption (57). Li et al. demonstrated in a mouse model of periodontitis that treatment with AMY-101, administered at a dose of 5 mg/kg, significantly decreased the RANKL/OPG ratio by reducing RANKL levels and increasing OPG expression (42). Furthermore, Kajikawa et al. demonstrated that AMY-101 significantly reduced the RANKL/OPG ratio in a non-human primate model of periodontitis. They observed that local administration of AMY-101 (0.1 mg/site) decreased RANKL expression and increased OPG levels (46). These modulation effectively suppressed osteoclast activity, leading to reduced bone resorption and preservation of alveolar bone integrity.

AMY-101 also demonstrates anti-inflammatory effects, further enhancing its therapeutic profile for periodontal disease. In addition to periodontal models, studies have shown that in human whole blood models, the use of AMY-101 (Cp40), can completely block complement activation and the secretion of pro-inflammatory cytokines in the immune system. Furthermore, blocking the complement receptor C3aR significantly reduces the levels of various cytokines, further demonstrating AMY-101’s potent inhibitory effect on complement activation and elevated inflammation levels. These findings underscore the potential of AMY-101 in treating periodontitis and various systemic inflammatory conditions (47). By inhibiting complement activation, AMY-101 effectively reduces inflammation, supports osteoblast differentiation, prevents bone loss, and promotes tissue regeneration.

## 5 The Effects of AMY-101 on periodontitis

### 5.1 *In vivo* study

In 2016, Maekawa et al. evaluated whether the C3 inhibitor Cp40 inhibits naturally occurring periodontitis in non-human primates (NHP) by administering intragingival injections of Cp40 once a week (5 animals) or 3 times a week (10 animals) for 6 weeks to NHPs with chronic periodontitis. Clinical periodontal examination and gingival sulcus fluid collection as well as gingival and bone biopsies were performed during the study period. Data were analyzed using one-way repeated measures analysis of variance. The results showed that Cp40 caused significant reductions in clinical indicators measuring periodontitis (gingival index and bleeding on probing), tissue destruction (probing pocket depth and clinical attachment level) or tooth mobility (48). The protective effect of Cp40 persisted for at least 6 weeks after discontinuation, demonstrating that Cp40 inhibits pre-existing chronic periodontitis and osteoclastogenesis in NHP, suggesting that it is a promising new adjunctive anti-inflammatory therapy for the treatment of human periodontitis. In 2018, Bostanci et al. (43) used a non-human primate (NHP) model to study the effects of Cp40, an AMY-101 derivative, in chronic periodontitis. Infected NHPs received Cp40 (0.5 mg/kg) injections once or thrice weekly for six weeks, followed by a six-week observation phase. Cp40 significantly reduced clinical indices, including gingival index (*p* < 0.05), bleeding on probing (*p* < 0.01), probing pocket depth (*p* < 0.01), and attachment loss (*p* < 0.01). The study linked these effects to the inhibition of the alternative complement pathway. Histological analysis showed reduced inflammatory cell infiltration and osteoclast activity, correlating with RANKL downregulation (49). These effects persisted for six weeks post-treatment, indicating Cp40’s sustained anti-inflammatory and bone-preserving efficacy. Additionally, Cp40 consistently lowered pro-inflammatory cytokines (TNF, IL-1β, IL-17a) while maintaining high levels of osteoprotegerin (OPG), highlighting its role as a C3 inhibitor for managing periodontal inflammation and preventing bone loss (50). Furthermore, Kajikawa et al. used an NHP model of chronic periodontitis to evaluate AMY-101 at a dose of 5 mg/kg, administered weekly for six weeks (37). This treatment significantly reduced probing depth (p < 0.01) and attachment loss (*p* < 0.01), with effects lasting up to eight weeks after the treatment ended. In a follow-up study, Hasturk et al. demonstrated AMY-101’s potential in bone preservation by administering 10 mg/kg twice weekly for four weeks in an NHP model (50). This regimen resulted in a 25% reduction in MMP-8 levels (*p* < 0.01) and decreased bone resorption markers, suggesting a protective effect on bone integrity.

### 5.2 Clinical study

In clinical studies, AMY-101’s safety and efficacy were further validated. Hajishengallis et al. (2021) conducted a Phase I safety study (NCT03316521), where AMY-101 was administered as single ascending doses ranging from 0.3 mg/kg to 15 mg/kg (51). This study confirmed AMY-101’s safety and tolerability across all dose levels, with no serious adverse events reported. In a subsequent Phase IIa trial in 2021, Hasturk et al. used a randomized, double-blind, split-mouth design to treat periodontitis patients with weekly injections of AMY-101 over three weeks (52). The study showed significant reductions in probing pocket depth and gingival bleeding index (p < 0.05), along with marked decreases in inflammation markers (MMP-8 and MMP-9), with no serious drug-related adverse events observed.

## 6 Future directions and conclusions

In previous studies, AMY-101, a novel complement inhibitor with potential for the treatment of periodontitis, has shown remarkable potential in the clinical treatment of periodontitis (52), effectively reducing inflammatory responses and tissue damage (53), improving periodontal health by inhibiting the C3 component of the complement system, and demonstrating a good safety and tolerability profile (**Table 1**). These results provide a solid foundation for the further development and clinical application of AMY-101. AMY-101’s selective inhibition of C3 effectively modulates the complement cascade, blocking the production of pro-inflammatory factors like C3a and C3b (6), which are central to the inflammation and tissue damage seen in periodontitis. The peptide’s cyclic structure enhances its stability and binding affinity to C3, promoting prolonged therapeutic effects (22). Furthermore, the complement inhibition achieved by AMY-101 not only alleviates local inflammation but also regulates bone regeneration (54). This comprehensive action on both inflammatory and regenerative pathways positions AMY-101 as a promising candidate for treating periodontitis and potentially other complement-driven inflammatory diseases.

**Table 1:** Dosage of AMY-101(CP40) for chronic periodontitis.

Author & Year	Type of study	Dosage	Frequency	Route of Administration	Results
Kajikawa T et al., 2017 (37)	NHP study	10 mg/kg	Once daily, for 28 days	Subcutaneous	Optimized dosing improved anti-inflammatory outcomes safely.
Bostanci N et al., 2018 (43)	NHP study	2 mg/kg	Daily for 7 days	Gingival injection	Significantly decreased inflammation while maintaining.
Hajishengallis G et al., 2019 (6)	Clinical Study	10 mg/kg	Daily for 2 weeks	Gingival injection	Demonstrated C3 inhibition with AMY-101 reduced gingival inflammation; well-tolerated.
Reis ES et al., 2019 (8)	NHP & Clinical Study	20mg/kg	Daily for 2 weeks	Subcutaneous	Confirmed AMY-101’s therapeutic potential with inflammation control and good safety.
Hasturk H et al., 2021 (52)	Clinical Study	0.1 mg per injection site, with two injection sites	Once a week for 3 weeks (on days 0, 7, and 14)	Gingival injection	Significant reduction in inflammation with no severe adverse effects observed.

However, the evidence is still insufficient. Larger clinical trials are needed to validate the efficacy and safety of AMY-101 in different populations. There is also a need to investigate the long-term effects of AMY-101 and to assess its long-term safety and efficacy in patients with chronic periodontitis. Second, studies should further investigate the optimal dosing regimen and dose of AMY-101 (55). These studies will provide an important reference for individualized treatment and help physicians to develop the most appropriate treatment regimen according to the patient’s specific situation (56). Third, AMY-101 has not yet been evaluated in combination with standard periodontal treatments, such as scaling and root planning (SRP), necessitating studies to explore potential synergistic effects. In addition, the mechanism of action of AMY-101 needs to be further investigated. Although current studies have established the principle that AMY-101 reduces inflammation and tissue damage by inhibiting C3 activation (57), its specific cellular and molecular pathways remain to be elucidated. Understanding AMY-101’s mechanism of action will enable scientists to develop more effective complement inhibitors and optimize the use of existing drugs, which also paves the way for further research and discussion on this topic.
